# Supra-Pubic Operation for Stone in the Bladder of a Girl Seven Years of Age

**Published:** 1866-08

**Authors:** W. F. Westmoreland

**Affiliations:** Professor of Surgery in the Atlanta Medical College


ARTICLE II.

*Supra-pubic Operation for Stone in the Bladder of a Girl seven years of age.* By W. F. WESTMORELAND, M. D., Professor of Surgery in the Atlanta Medical College.

In March, 1862, I was requested by Mr. G—— to visit his daughter, seven years of age, who, he said, had for several years suffered from stone in the bladder. Upon my arrival, I found my little patient poorly developed for her age—delicate and considerably emaciated from long continued suffering. From the history of the case, as given by the mother, I learned that she had suffered for three or four years from the ordinary symptoms of stone in the bladder; and that, for three or four years previous to my visit, her sufferings had been intense, and was evidently telling upon her constitution.

Upon examination with the sound, a stone of considerable size was readily detected. It was evident that the immediate removal of the calculus was the only means of relieving the sufferings and prolonging the life of the patient.

The propriety of an immediate operation decided upon, the question presented itself as to the best method of removing the stone. The dilitation of the urethra, either with or without the longitudinal section of this canal, as recommended in the adult female by several surgeons for the removal of stone through this natural opening from the bladder, was rejected as impracticable in one so young; that if it was possible, it would almost certainly entail that terrible condition, permanent incontinence of urine. "Vaginal Litholomy"—which in the adult may be performed with less risk, perhaps, to life than any other operation for stone in the bladder in the female—was regarded as impracticable in one so young and poorly developed as our little patient, requiring a length of time to dilate the vaginal canal—entailing an amount of suffering and irritation, which, in her condition, she was poorly able to bear; and at last, if successful in removing the stone by this procedure, a vesico-vaginal fistula being almost the invariable result, would require a second operation, which, in a subject only seven years old, would be attended with many difficulties.

Lithotripsy, the method, perhaps, the most frequently resorted to for the removal of stone from the female bladder, was regarded as difficult and hazardous. In addition to the tender age of the patient, from long continued irritation, the urethra and adjacent tissues were considerably indurated, and this canal consequently constricted. The bladder, too, was so excessively irritable that it was found impossible to have retained in this viscus the least quantity of water, so that if we could have succeeded in overcoming the first difficulty, and introduced the lithotrite into the bladder, we would have experienced, perhaps, a greater difficulty in attempting to grasp the stone in the flaced condition of the organ, and more danger still by lacerating and contusing the mucous membrane with the instrument, if we had attempted to crush it.

In consultation with Drs. H. W. Brown and N. D'Alvigny, the *Supra-pubic* or *high* operation was determined upon. In a few days after my first visit, assisted by the above mentioned gentlemen, the following operation was performed:

An incision two inches and a half or three inches long was made through the skin and cellular tissue, commencing above and following the linea alba, and terminating at the symphysis; a partial section of the abdominal parieties was next made. As it was impossible, from the irritable condition of the bladder, to distend it with fluids, and from the irritable and small size of the urethra, to introduce the sound of M. Come, an ordinary metallic bougie was now introduced and the contracted bladder forced above the symphysis pubis by the instrument. A careful dissection was now made until the bladder was reached; by means of two tenaculums inserted in the walls of the bladder, the fourth of an inch apart, the organ was fully under the control of the assistant who held the two hooks. The walls of the bladder were now punctured between the two tenaculums, and the incision made sufficiently large to introduce the forceps.

A stone an inch by three-quarters of an inch in diameter was, without difficulty, removed with the forceps.

A small silver catheter was now introduced into the urethra and confined in the bladder. For the first eight or ten days, there was considerable febrile excitement. I found great difficulty, after the first twenty-four hours, in retaining a catheter in the bladder—the presence of the instrument causing great uneasiness.

On the twelfth day after the operation, the battle of Shiloh was fought, and I was immediately ordered to Corinth, Mississippi. Upon my return to Atlanta in June following, I was gratified to find my little patient entirely recovered.

Dr. D'Alvigny, in whose care the patient was left, informed me that she had considerable fever, with diarrhœa, for several days after I left; in every other particular did well.

I last saw the patient two years after the operation, and she was then in the most perfect health.

